# *SYN1* variant causes X-linked neurodevelopmental disorders: a case report of variable clinical phenotypes in siblings

**DOI:** 10.3389/fneur.2024.1359287

**Published:** 2024-03-21

**Authors:** Bin Ren, Xiaoyan Wu, Yuqiang Zhou, Lijuan Chen, Jingzi Jiang

**Affiliations:** ^1^Shanghai Nyuen Biotechnology Co., Ltd., Shanghai, China; ^2^Department of Neurology, Affiliated Hospital of Guilin Medical University, Guilin, China

**Keywords:** *SYN1*, reflex epilepsy, bathing epilepsy, genotype–phenotype correlation, clinical heterogeneity

## Abstract

The *SYN1* gene encodes synapsin I, variants within the *SYN1* gene are linked to X-linked neurodevelopmental disorders with high clinical heterogeneity, with reflex epilepsies (REs) being a representative clinical manifestation. This report analyzes a Chinese pedigree affected by seizures associated with *SYN1* variants and explores the genotype–phenotype correlation. The proband, a 9-year-old boy, experienced seizures triggered by bathing at the age of 3, followed by recurrent absence seizures, behavioral issues, and learning difficulties. His elder brother exhibited a distinct clinical phenotype, experiencing sudden seizures during sleep at the age of 16, accompanied by hippocampal sclerosis. Whole exome sequencing (WES) confirmed a pathogenic *SYN1* variant, c.1647_1650dup (p. Ser551Argfs*134), inherited in an X-linked manner from their mother. Notably, this variant displayed diverse clinical phenotypes in the two brothers and one previously reported case in the literature. Retrospective examination of *SYN1* variants revealed an association between truncating variants and the pathogenicity of REs, and non-truncating variants are more related to developmental delay/intellectual disability (DD/ID). In summary, this study contributes to understanding complex neurodevelopmental disorders associated with *SYN1*, highlighting the clinical heterogeneity of gene variants and emphasizing the necessity for comprehensive genetic analysis in elucidating the pathogenic mechanisms of such diseases.

## Introduction

Reflex epilepsies (REs) are characterized by recurrent seizures consistently triggered by specific stimuli, such as light, music, hot water, and bathing (1). Limited research has been conducted on the genetic underpinnings of REs, with *SYN1* being one of the few identified causative genes in humans (2, 3). *SYN1* encodes synapsin I, a neuronal phosphoprotein that regulates axonogenesis and synaptogenesis (4). *SYN1* gene variants have been implicated in two X-linked disorders: epilepsy with variable learning disabilities and behavior disorders (EPILX, [MIM:300491]) and intellectual developmental disorder 50 (XLID50, [MIM:300115]). EPILX manifests as epileptic seizures, learning difficulties, autism spectrum disorders, aggressive behavior, behavioral abnormalities, or variable developmental delays (2, 5–7). In contrast, XLID50 is characterized by varying degrees of intellectual disability, autistic features, and mild brain malformations, typically without seizures (6, 8–10).

The disease phenotype associated with *SYN1* variants is highly heterogeneous, with individuals within the same family carrying identical genetic variants displaying a broad spectrum of clinical manifestations and varying degrees of severity (5, 6, 11). REs, a distinctive clinical hallmark, are frequently observed in individuals with *SYN1* variants. Seizures are mostly triggered by water exposure (bathing, showering, swimming), but other stimuli like tooth brushing, rubbing with a towel, fever, and so on have been reported (4–7, 12, 13). Though recent reports suggest a tendency for seizures, especially REs to be more prevalent in individuals with truncating variants, while non-truncating variants are associated with developmental disorder (6), definitive genotype–phenotype are yet to be established and require intensive investigation.

In this context, our study aims to contribute valuable insights into the genotype–phenotype correlations of *SYN1*-related disorders through a Chinese pedigree with two male patients and literature Review. In this family, two brothers were identified with *SYN1* truncating variants, and displayed different clinical features. Additionally, our study involves a comprehensive review of the pathogenicity of truncating and non-truncating variants from existing literature, laying the foundation for future in-depth investigations into *SYN1*-related disorders.

## Materials and methods

### Subjects

A 9-year-old boy (proband, II-1) and his elder brother (II-2) with epilepsy were admitted at Affiliated Hospital of Guilin Medical College. Whole exome sequencing (WES) was performed on both patients. The parents of the siblings were also enrolled in this investigation to elucidate the genetic inheritance pattern. This study was approved by the Institutional Research Ethics Committee of the Affiliated Hospital of Guilin Medical College (Ethical Approval Number:2022QTLL) and written informed consent was obtained from the parents of the pedigree.

### Whole exome sequencing and data analysis

Genomic DNA was extracted from whole blood samples for library preparation using the MagPure Tissue&Blood DNA LQ Kit (Magen). The T345V1 Exome Research Panel probes (iGeneTech, Shanghai, China) were utilized to capture the exon region following the manufacturer’s recommendations. The raw data was sequenced on NovaSeq6000 platforms (Illumina). Reads were mapped to the hg19 reference genome using BWA MEM (v0.7.17). PCR and optical duplicate marking were conducted using GATK (v4.1.4.0), and local realignment around indels and base quality score recalibration were carried out by GATK (v3.8.1). Finally, variants were identified using GATK HaplotypeCaller 1 (v3.8.1) and filtered by bcftools with the following criteria (v1.9): mapping quality≥40; depth ≥ 4.

We prioritized the rare variants with a minor allele frequency < 0.005 in Genome Aggregation Database (gnomAD),^1^ and Exome Aggregation Consortium. We retained potentially pathogenic variants, including frameshift, nonsense, canonical splice site, initiation codon, in-frame, and missense variants. Variants were further filtered based on epilepsy-associated gene model and possible inheritance models. Candidate variants were confirmed and co-segregation studies were performed using Sanger sequencing. Candidate variants were analyzed according to the American College of Medical Genetics and Genomics (ACMG) guidelines (14).

### Sanger sequencing

Sanger sequencing was performed on the two brothers and their parents to confirm the candidate variant identified in *SYN1* through WES analysis. The primer design was accomplished using Primer Premier 5 and synthesized by MapBioo (Shanghai, China). The forward and reverse primer sequences were as follows: Forward: 5′-TTCCAAGTCCCACCTCAGCG-3′; Reverse: 5′-TTGGCGGAGCCGGGCCAGA-3′. Amplified products underwent sequencing utilizing an ABI 3730 DNA sequencer (Applied Biosystems).

## Results

### Clinical overview of the pedigree

The proband (II-1), initially manifested loss of consciousness at the age of 3, which was triggered by bathing in a bathtub filled with lukewarm water (approximately 37°C). The episodes included loss of consciousness, perioral cyanosis and hypotonia, and lasted for approximately 1 min before consciousness was regained. Subsequently, several seizures occurred specifically during bathing. To mitigate this, bathing in the bathtub was discontinued. The patient’s mother adapted by using a warm wet towel to cleanse him, a measure that successfully averted any subsequent seizure activity. Notably, after a hiatus of 4–5 months, the child was able to return to his regular routine of bathing without experiencing any further episodes of seizures. Commencing at age 6, he exhibited recurring absence epilepsy episodes, spontaneously resolving within seconds, but occurring as frequently as daily, particularly during homework sessions. Interictal electroencephalography (EEG) disclosed 2–3 Hz sharp slow wave discharges, notably in the left occipital and middle/posterior temporal regions during sleep, along with sharp wave discharges in the right anterior temporal lobe on another EEG test. The patient reported experiencing episodes of staring and the ictal EEG showed high amplitude spike-and-wave activity and sharp-and-slow wave activity across multiple leads, with both long and short bursts occurring. Magnetic resonance spectroscopy (MRS) analysis showed metabolic abnormalities in the hippocampal head on both sides, the left body, and the right tail, suggesting neuronal loss and gliosis. Additionally, the proband displayed abnormal behavioral and psychiatric features, including uncontrolled temper (especially during homework), attention deficit hyperactivity disorder (ADHD), and learning difficulties. The patient initially received treatment with 125 mg of magnesium valproate sustained release tablets twice a day, which reduced the frequency of absence seizures. After 2 weeks, the dosage was adjusted to 250 mg twice a day. Following this adjustment, the frequency of absence seizures during homework periods further diminished from 4 to 5 times daily to 1–2 times daily. As the seizures had minimal impact on the patient’s daily life, it was decided to maintain the current treatment plan without any immediate changes.

Reflex epilepsies, such as hot water epilepsy and bathing epilepsy, are characterized by seizures triggered by specific stimuli, particularly involving water during bathing. Hot water epilepsy, a well-known variety that is frequent in South India, manifests a few key clinical characteristics: (1) triggering of seizures by pouring hot water, with temperatures ranging from 38°C to 55°C, over the head; (2) the absence of neurodevelopmental problems or detectable abnormalities in neuro-imaging for most affected children; (3) generally favourable outcome for the majority of children diagnosed with hot water epilepsy (15–19). But a less well-known infantile variety, primarily documented in France, exhibits several distinct clinical features: (1) Seizures commence before the age of 1 year, triggered by immersion in hot water at approximately 37.5°C; (2) Similar to the variant prevalent in South India, these children typically do not exhibit neurodevelopmental problems or abnormalities in neuro-imaging; (3) The prognosis for these children is also generally positive; (4) EEGs often reveal a focal onset of seizures, predominantly in the temporal region; (5) No other type of seizure was reported (20). The phenotypes of our patient do not align with the characteristics of the aforementioned diseases. Bathing epilepsy, in contrast, is characterized by seizures that can be triggered by immersion in water at temperatures close to or below the normal body temperature, and some patients may experience neurological symptoms (2, 18, 21, 22). The diagnosis of bathing epilepsy is supported by the proband’s clinical presentation, which more closely meets the diagnostic criteria for this condition.

His elder brother (II-2), now aged 16, exhibited normal developmental history and intelligence until experiencing a sudden epileptic seizure during sleep for 3 times in 1 month at the age of 16, characterized by loss of consciousness and teeth clenching, with no identifiable trigger. Ambulatory EEG revealed dispersed epileptic discharges during sleep, predominantly in the left occipital region, without any clinical events occurring during monitoring. MRS demonstrated bilateral hippocampal sclerosis and abnormal neurometabolic profiles indicative of neuronal loss and gliosis. Antiepileptic therapy with magnesium valproate sustained release tablets (250 mg twice daily) successfully prevented further seizure for 2-year follow-up.

The EEG of the two brothers show some notable similarities. Both individuals display epileptiform discharges, which are more prominent on the left side and tend to occur during sleep. Interestingly, the brother exhibits a broader distribution of epileptiform discharges compared to the proband. The consistency across their EEG and MRS results strengthens the likelihood of a shared etiology behind their neurological presentations.

The two brothers were the only children in their family. No family history of neurological disorders, behavioral issues, or febrile seizures was reported. Both parents were healthy and nonconsanguineous.

### Genetic analysis

**Figure 1:**
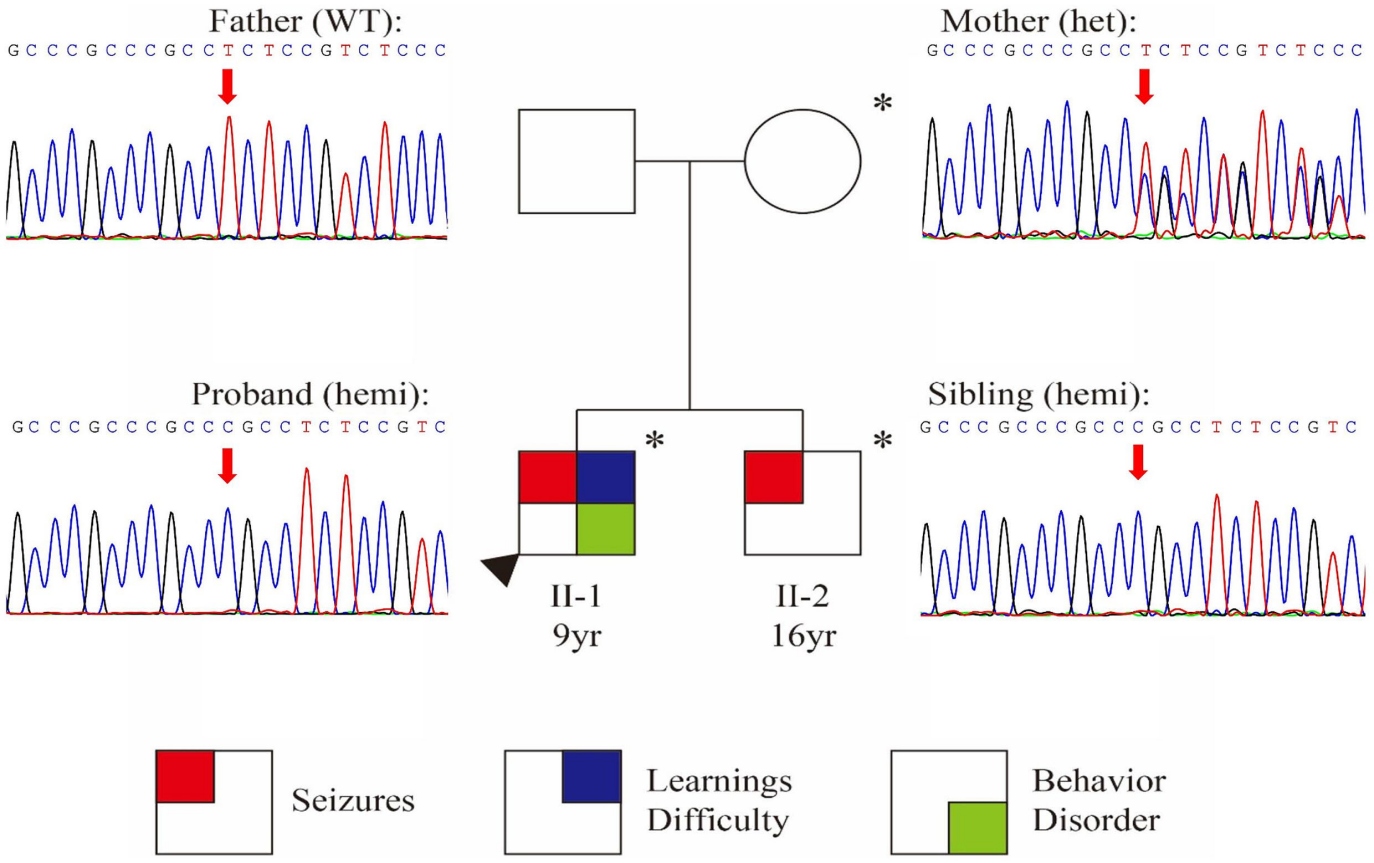
Identification of a frameshift variant in *SYN1*. Pedigree chart and Sanger confirmation of the family. Individuals with the *SYN1* variant are represented with an asterisk (*) symbol.

Whole Exome Sequencing identified a frameshift variant in *SYN1* [NM_006950.3:c.1647_1650dup (p.Ser551Argfs*134)] in both brothers (Figure 1). Sanger sequencing confirmed maternal inheritance of the variant (Figure 1). The X-linked pattern of segregation was observed, with affected siblings being hemizygous, unaffected mother being heterozygous and father being wild-type (father; PP1). The variant c.1647_1650dup (p.Ser551Argfs*134) is predicted to cause a frameshift that could potentially result in nonsense-mediated mRNA decay (PVS1) (23). The variant was absent in gnomAD v4.0.0 databases (PM2_Supporting). The variant was classified as conflicting classifications of pathogenicity in ClinVar with “Pathogenic” and “Uncertain significance”(Variation ID:1994692). Based on the above evidence, this variant can be classified as pathogenic (PVS1 + PM2_Supporting+PP1) according to ACMG guidelines (14, 23, 24).

### Clinical features of individuals with *SYN1* c.1647_1650dup (p. Ser551Argfs*134) variant

The c.1647_1650dup (p.Ser551Argfs*134) variant was previously reported in a boy with seizures, global developmental delay, mild intellectual disability, autism spectrum disorder, motor stereotypies, attention-deficit/hyperactivity disorders, who experienced reflex seizures after bathing, showering, fingernail clipping (2). Including our patients, c.1647_1650dup has been reported in three patients with detailed records available, but the clinical phenotypes were highly variable, even the two brothers in the same family. All the three cases demonstrated a favorable response to antiepileptic drugs. In the case reported by Accogli, the affected individual achieved seizure freedom following treatment with Oxcarbazepine, Sulthiame, Valproate, and Lamotrigine (2).

In addition, this variant has been submitted three times in ClinVar database, with each submission coming from patients identified through clinical testing. The patient submitted by Invitae was diagnosed as “epilepsy, X-linked 1, with variable learning disabilities and behavior disorders,” while the patients submitted by Baylor Genetics were diagnosed with “intellectual disability, X-linked 50” and “Epilepsy, X-linked 1, with variable learning disabilities and behavior disorders.” These phenotypes align with those related to *SYN1* disease. However, without specific clinical information, it’s unclear whether they experience reflex seizures or how they respond to anti-epileptic drugs, which inhibits further analysis of clinical variations.

### Classification of *SYN1* variants

**Figure 2:**
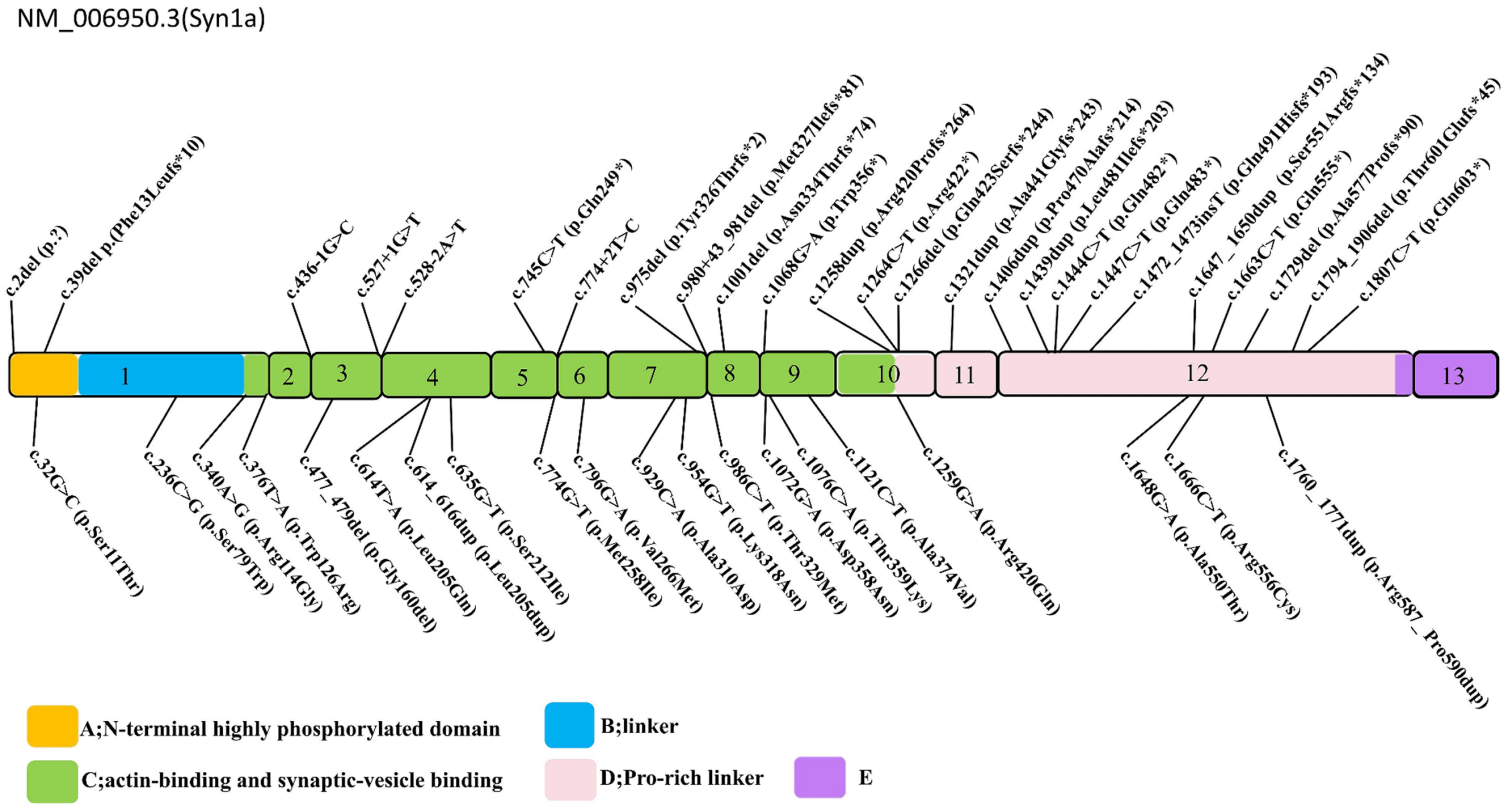
Schematic representation of the *SYN1* gene. Exons 1–13 are depicted, truncating variants depicted above, non-truncating variants depicted below, the variants Thr567Ala, Ala51Gly, and Gly240Arg are removed. The conserved A (N-terminal highly phosphorylated domain), B (linker), and C (functional domain) domains are depicted in yellow, blue, green, respectively; the D, E domains in pink, and purple.

The spectrum of *SYN1* gene variants is extensive, encompassing truncating variants such as frameshift, nonsense, splice-site, and start-loss variants, alongside non-truncating variants, including missense alterations, in-frame deletion, and in-frame insertion (Figure 2). Syn1 knockout mice have been observed to display an epileptic phenotype (25, 26), which is similar to the phenotype seen in human patients. Functional studies have also confirmed that truncating variants of *SYN1* can impair synaptic function (9, 27). According to ClinGen dosage sensitivity curation standard operating procedures (23), the evidence supporting haploinsufficiency in *SYN1*-related X-linked complex neurodevelopmental disorder is substantial, establishing loss of function as the pathogenic mechanism for *SYN1*-related disorders. *SYN1* truncating variants were reported in 62 patients (Table 1), with a total of 25 different variants. All 25 variants were loss of function variants, classified as likely pathogenic or pathogenic (Figure 2). Truncating variants introduce a premature termination codon, leading to nonsense-mediated mRNA decay or generating a truncated protein that lacks phosphorylation sites (27–29). These alterations disrupt synaptic vesicle transport, increasing network excitability and firing/bursting activity (9, 27, 30).

**Table 1:** Clinical and genetic characteristics of patients with *SYN1*-related disorders in literature and the study.

Patient ID	Variant	Sex	Trigger of RE	Other seizure type	EEG	ASMs	Response to medications	Development	Other phenotypes	Study
1	c.1647_1650dup (p.Ser551Argfs*134)	M	During bathing	Recurrent absence epilepsy at 6y	Interictal EEG:2–3 Hz sharp slow waves discharges in temporal and occipital; ictal EEG: high amplitude spike-and-wave activity and sharp-and-slow wave activity.	Magnesium Valproate	Decreased Seizure frequency	Learning difficulty, uncontrolled tempers, ADHD	-	The present study
2	c.1647_1650dup (p.Ser551Argfs*134)	M	-	GTCS at 16y	Interictal EEG: Abnormal discharge in bilateral occipital area, especially in left.	Magnesium Valproate	Seizure-free	Normal	-	The present study
3	c.152C > G (p.Ala51Gly)/c.1699A > G (p.Thr567Ala)	M	-	-	NA	-	-	ASD, mild ID	NA	Fassio et al. (9)
4	c.1699A > G (p.Thr567Ala)	M	-	-	NA	-	-	ASD	NA	Fassio et al. (9)
5	c.1648G > A (p.Ala550Thr)	M	-	-	NA	-	-	ASD	NA	Fassio et al. (9)
6	c.1648G > A (p.Ala550Thr)	F	NA	Idiopathic partial epilepsy	NA	NA	NA	ASD, mild ID	NA	Fassio et al. (9)
7	c.1068G > A (p.Trp356*)	M	-	TCS from the age of 16 years	NA	NA	NA	Normal	Macrocephaly, Magnetic resonance imaging (MRI) showed mild generalized cerebral and cerebellar atrophy in keeping with his age	Garcia et al. (11)
8	c.1068G > A (p.Trp356*)	M	-	Tonic–clonic seizures until aged 7	NA	NA	NA	Normal	Cerebrovascular accident	Garcia et al. (11)
9	c.1068G > A (p.Trp356*)	M	-	GTCS between the ages of 11 years and 18 years.	Spike focus in the left temporal region.	NA	NA	IQ 72, learning difficulties, extreme physical aggression	-	Garcia et al. (11)
10	c.1068G > A (p.Trp356*)	M	-	Had first fit at 18 years of age and has continued to have occasional seizures	NA	NA	NA	Normal	-	Garcia et al. (11)
11	c.1068G > A (p.Trp356*)	M	-	-	Normal	NA	NA	Learning difficulties	Macrocephaly, episodic aggressive outbursts	Garcia et al. (11)
12	c.1068G > A (p.Trp356*)	M	-	Began to have partial and complex-partial seizures at the age of 16.	Normal	NA	NA	Normal	repetitive stereotyped behaviors, fits that consist of episodes of jerking of his left leg; these sometimes develop into jerking of his left leg and arm followed by unconsciousness.	Garcia et al. (11)
13	c.1068G > A (p.Trp356*)	M	During bathing	-	Normal	NA	NA	Normal	-	Garcia et al. (11)
14	c.1068G > A (p.Trp356*)	M	-	Diagnosed as having nocturnal epilepsy at the age of 6	Bilateral non-specific changes	NA	NA	Normal	-	Garcia et al. (11)
15	c.1068G > A (p.Trp356*)	M	-	-	Normal	NA	NA	ASD, moderate learning difficulties, severe aggression behavior	Macrocephaly	Garcia et al. (11)
16	c.1663C > T (p.Gln555*)	M	-	Complex partial seizures	Normal	PHT + CBZ + Pb	~2/mo	NA	NA	Nguyen et al. (5)
17	c.1663C > T (p.Gln555*)	M	-	Complex partial seizures	NA	NA	NA	NA	NA	Nguyen et al. (5)
18	c.1663C > T (p.Gln555*)	M	-	Complex partial seizures	NA	NA	NA	NA	NA	Nguyen et al. (5)
19	c.1663C > T (p.Gln555*)	M	After face rubbing with wet towel or showering	FS, complex partial seizures	Ictal EEG:R temporal rhythmic theta	PHT, CLB, LTG	Seizure control was good but not perfect with CLB (30 mg/day). LTG monotherapy (200 mg/day): Seizure-free for the last 6 years.	IQ:78	Mild R hippocampal atrophy, Dyslexia	Nguyen et al. (5)
20	c.1663C > T (p.Gln555*)	M	After bath/shower	Complex partial seizures	Normal	LTG	Seizure-free	IQ:79	Dyslexia	Nguyen et al. (5)
21	c.1663C > T (p.Gln555*)	M	During shower or while testing temperature of shower/faucet water	Rare complex partial seizures, GTC	Ictal EEG:Late diffuse rhythmic theta	LTG	Seizure-free	Normal	-	Nguyen et al. (5)
22	c.1663C > T (p.Gln555*)	M	After bath/swimming	Occasional complex partial seizures	NA	NA	NA	NA		Nguyen et al. (5)
23	c.1663C > T (p.Gln555*)	M	After shower/bath	Occasional complex partial seizures	Normal	LTG, LEV	Rare seizures	IQ:78		Nguyen et al. (5)
24	c.1663C > T (p.Gln555*)	M	During bath and nail clipping	Occasional complex partial seizures	Normal	LTG + CBZ	Seizure-free	PDD, mixed language deficits	Mild left hemispheric atrophy (including hippocampus)	Nguyen et al. (5)
25	c.1663C > T (p.Gln555*)	M	After shower/bath	Complex partial seizures	Normal	CBZ	Seizure-free	PDD, mixed language deficits		Nguyen et al. (5)
26	c.1663C > T (p.Gln555*)	F	-	FS	NA	-	-	-	Mild dyslexia	Nguyen et al. (5)
27	c.1663C > T (p.Gln555*)	F	-	FS	NA	-	-	Normal	-	Nguyen et al. (5)
28	c.236C > G (p.Ser79Trp)	M	-	-	-	-	-	moderate ID, severe perseveration and echolalia	-	Guarnieri et al. (8)
29	c.236C > G (p.Ser79Trp)	M	-	-	-	-	-	DD, ID	-	Guarnieri et al. (8)
30	c.236C > G (p.Ser79Trp)	F	-	-	-	-	-	ID	-	Guarnieri et al. (8)
31	c.236C > G (p.Ser79Trp)	M	-	-	-	-	-	DD, ID	-	Guarnieri et al. (8)
32	c.1264C > T (p.Arg422*)	M	Triggered by defecation; After age 5, seizures tended to occur during or soon after showering	Nocturnal epilepsy	EEG at age 5 years showed left temporal spikes Interictal EEG was significant for generalized-appearing spikes	CZP	Reduce seizures; seizures were reduced by 40 to 50% after VNS, His reflex bathing seizures were not significantly improved with the addition of VNS.	ID, autism spectrum disorder	-	Sirsi et al. (7)
33	c.527 + 1G > T	M	Occurring especially after hot water had been poured on his body	Nonreflex seizures consisting of a tingling ascending sensation starting from the lower limbs	Ictal video EEG characterized by bilateral rhythmic theta activity over the frontocentral and vertex regions.	NA	NA	Learning disability involving reading, writing, and calculation	-	Peron et al. (13)
34	c.527 + 1G > T	M	Occurring especially after hot water had been poured on his body	NA	NA	NA	NA	Mild ID	-	Peron et al. (13)
35	c.1264C > T (p.Arg422*)	M	After showering, rubbing with towel, watching his sister having a shower	Nocturnal tonic–clonic seizures at 6 y	EEG interictal:right temporal, left anterior temporal.	CLB, VPA	Decreased seizures frequency	Speech delay, aggressive behavior, ADHD	-	Accogli et al. (2)
36	c.1439dup (p.Leu481Ilefs*203)	M	During or after bathing	-	EEG interictal:left frontotemporal.	CLB, VGB, CBZ, CLB	Partial response	Speech delay, hyperactivity	-	Accogli et al. (2)
37	c.774 + 2 T > C	M	During or after showering, rubbing with towel	-	EEG interictal:right central, temporal	CLB	Decreased seizures frequency	GDD, moderate ID, ASD, motor stereotypies, aggressive behavior, echolalia	-	Accogli et al. (2)
38	c.436-1G > C	M	During showering	-	EEG interictal:Normal.	NA	NA	Normal	NA	Accogli et al. (2)
39	c.1406dup (p.Pro470Alafs*214)	M	During bathing	Focal impaired awareness seizures, 2.5 y	EEG interictal:Bilateral temporal	VPA	Poor response	Mild GDD, speech delay, ADHD	-	Accogli et al. (2)
40	c.1406dup (p.Pro470Alafs*214)	M	During bathing	Focal impaired awareness seizures, 2 y	EEG interictal:Bilateral temporal	VPA	Poor response	Mild GDD, speech delay, ADHD	-	Accogli et al. (2)
41	c.1406dup (p.Pro470Alafs*214)	F	During bathing	Focal impaired awareness seizures, 9 m	EEG interictal:Theta activity over the right temporal regions.	VPA	Seizure-free	Mild GDD, speech delay, autistic features	-	Accogli et al. (2)
42	c.1472_1473insT (p.Gln491Hisfs*193)	M	After bathing/showering, haircutting, fingernail clipping, watching someone while bathing, idea of bathing	Nocturnal autonomic seizures, at 5y	EEG interictal:Bilateral centrotemporal; Ictal-video EEG:onset of seizure with initial theta high-voltage polymorphic activity over the frontal-temporal region.	VPA, STM, LTG	No	GDD, moderate ID, ASD, ADHD	-	Accogli et al. (2)
43	c.929C > A (p.Ala310Asp)	M	During or after bathing, hair washing	Nocturnal autonomic seizures, 1 y 3 mo; febrile seizures at 3y	EEG interictal:Normal; Ictal-video EEG:high-voltage polymorphic theta activity over the right frontal-temporal area.	CBZ	Seizure-free, avoidance of warm water	Normal	-	Accogli et al. (2)
44	c.1647_1650dup (p.Ser551Argfs*134)	M	After bathing, showering, fingernail clippling	Nocturnal autonomic seizures, tonic–clonic seizures status epilepticus, 7 y	EEG interictal:right and left temporal; Ictal-video EEG:rtythmic theta activity over the left temporal.	OXC, STM, VPA, LTG	Seizure-free	GDD, mild ID, ASD, motor stereotypies ADHD	-	Accogli et al. (2)
45	c.1760_1771dup (p.Arg587_ Pro590dup)	F	During or after bathing/showering	Infantile spasms, 8 months; tonic–clonic seizures with automatism, 2 y; atonic atypical absence seizures	EEG interictal:Bursts of slow spike–wave; Ictal-video EEG:beta diffuse.	LTG, VPA, VGB, LAC, LEV, BRV, ZNS, steroids, KD, CBL, RUF	Decreased seizure frequency CBL, RUF, BRV	GDD, severe ID, ADHD	-	Accogli et al. (2)
46	c.1266del (p.Gln423Serfs*244)	M	During immersion of the feet in water and during febrile events illnesses	Febrile seizure between 9 and 18 months, Focal to bilateral tonic–clonic seizures; nocturnal tonic–clonic seizures in cluster	EEG interictal:Twice, normal in adulthood.	CBZ, LTG, VPA	VPA and avoidance of warm water on his feet	Mild GDD, speech delay, mild ID, autistic traits	-	Accogli et al. (2)
47	c.1076C > A (p.Thr359Lys)	M	-	TCS triggered by fever/ febrile seizures	Normal	-	-	Profound GDD, motor delay, language development delay, social interaction problems	Bilateral esotropia	Xiong et al. (4)
48	c.1444C > T (p.Gln482*)	M	-	TCS	video EEG revealed occasional sharp-waves in the bilateral frontal areas during sleep, that was absent in wake-time.	Levetiracetam	Well controlled	ID, aggressive behaviors, hyperactivity, attention deficit	ametropia	Xiong et al. (4)
49	c.1807C > T (p.Gln603*)	M	Tooth brushing	Spontaneous GTCS during sleep.	interictal EEG showed intermittent rhythmic slow waves in the left frontotemporal region.	Oxcarbazepine	Seizure-free for 1 year follow up.	Normal	-	Zhou et al. (12)
50	c.1807C > T (p.Gln603*)	M	Tooth brushing	-	NA	-	-	Mild learning difficulties during childhood	-	Zhou et al. (12)
51	c.1807C > T (p.Gln603*)	M	-	-	NA	-	-	Aggressive behaviors, learning difficulties	-	Zhou et al. (12)
52	c.796G > A (p.Val266Met)	M	-	-	NA	-	-	Mild ID, autistic features, paranoid schizophrenia	-	Ibarluzea et al. (27)
53	c.1259G > A (p.Arg420Gln)	M	-	-	NA	-	-	ID from early childhood, mental regression, autistic features	Abnormal eye contact and language problem, sphincter dysfunction, marked generalized frontal atrophy in brain MRI	Darvish et al. (10)
54	c.1259G > A (p.Arg420Gln)	M	-	-	NA	-	-	ID from early childhood, mental regression, autistic features	Abnormal eye contact and language problem, sphincter dysfunction, marked generalized frontal atrophy in brain MRI	Darvish et al. (10)
55	c.635G > T (p.Ser212Ile)	M	-	-	Normal	-	-	ASD	NA	Rossi et al. (26)
56	c.2del (p.?)	M	Rubbing with towel, defecation	Nocturnal focal seizures	Normal	Sultiame, Oxcarbazepine	Pharmo resistance	ID, behavioral issues, ADHD,	NA	Parenti et al. (6)
57	c.2del (p.?)	M	Warm bath	Dizzy spells	Normal	NA	NA	ID, behavioral issues	NA	Parenti et al. (6)
58	c.2del (p.?)	M	-	Notices seizure beforehand, occurrence approx. Every 6 months, stiffness—lasting a few seconds—and salivation	NA	Valproate, Levetiracetame	NA	DD, ID, behavioral issues, ASD	NA	Parenti et al. (6)
59	c.1258dup (p.Arg420Profs*264)	M	-	Focal seizures (nocturnal +++)	Abnormal background rhythm of the more or less pointed theta type, perhaps with a central predominance, without any real paroxysm	TRILEPTAL	Seizure-free	DD, ID, behavioral issues, ASD	NA	Parenti et al. (6)
60	c.975del (p.Tyr326Thrfs*2)	M	Emotions and lightning	Generalized tonic–clonic seizures	Multifocal slow activity	Depakine/Sabril/Lamictal	1–2 seizures/year with LAMICTAL	DD, ID, behavioral issues, ASD	NA	Parenti et al. (6)
61	c.975del (p.Tyr326Thrfs*2)	M	-	Myoclonia Isolated	Interictal generalized spike wave activity	Micropakine	Seizure-free	Behavioral issues, ADHD	NA	Parenti et al. (6)
62	c.1729del (p.Ala577Profs*90)	M	Bathing, sleep, illness, digestive troubles	Generalized tonic–clonic seizures, febrile seizures	Frontal (central) right	Vimpat Fycompa, Lamictal (Tritherapy)	Pharmo resistance	DD, ID, behavioral issues, ASD	NA	Parenti et al. (6)
63	c.1001del (p.Asn334Thrfs*74)	M	-	Tonic–clonic generalized	Left fronto-temporal focus without clinical manifestation	Valproate + lamotrigin	Seizure-free or reduced	DD, ID, behavioral issues, ASD	NA	Parenti et al. (6)
64	c.1001del (p.Asn334Thrfs*74)	M	-	Tonic–clonic generalized	NA	Valproate	NA	DD, ID	NA	Parenti et al. (6)
65	c.1001del (p.Asn334Thrfs*74)	M	NA	NA	NA	NA	NA	DD, ID, behavioral issues	NA	Parenti et al. (6)
66	c.1001del (p.Asn334Thrfs*74)	M	NA	Epilepsy	NA	NA	NA	DD, ID	NA	Parenti et al. (6)
67	c.1439dup (p.Leu481Ilefs*203)	M	-	Tonic–clonic seizures	NA	Tegretol	Seizure-free	DD, ID	NA	Parenti et al. (6)
68	c.1794_1906del (p.Thr601Glufs*45)	M	-	Tonic–clonic seizures and focal seizures with impaired awareness	Slow focus, posterior left	Epitomax, keppra, rivotril	Pharmo resistance	DD, ID, behavioral issues, ASD	NA	Parenti et al. (6)
69	c.1447C > T (p.Gln483*)	M	Shower with warm water	Focal seizures	Normal	Oxcarbamazepinez, Brevetiracetam	Pharmo resistance	DD, behavioral issues, ADHD	NA	Parenti et al. (6)
70	c.1447C > T (p.Gln483*)	M	Shower with warm water	Focal seizures	Normal	Lamotrigine	Pharmo resistance	Behavioral issues	NA	Parenti et al. (6)
71	c.1447C > T (p.Gln483*)	M	Shower with warm water	Focal seizures	Normal	Lacosamide	Pharmo resistance	NA	NA	Parenti et al. (6)
72	c.1321dup (p.Ala441Glyfs*243)	M	Hot water	Epilepsy	NA	Valproate, lamotrigine, clobazam	Pharmo resistance	DD, ID	NA	Parenti et al. (6)
73	c.1072G > A (p.Asp358Asn)	M	-	Focal seizures with secondary generalization	Right occipital spikes	Carbamazepine	Seizure-free	DD, behavioral issues, ASD	NA	Parenti et al. (6)
74	c.986C > T (p.Thr329Met)	F	NA	-	NA	NA	NA	DD, behavioral issues	NA	Parenti et al. (6)
75	c.1264C > T (p.Arg422*)	M	Showering and using the swimming pool	Tonic–clonic seizures; also absence/focal impaired awareness seizures	Multifocal epileptiform activity (occipital, frontal)	Clonazepam, oxcarbazepine and valproate	Pharmo resistance	DD, ID	NA	Parenti et al. (6)
76	c.954G > T (p.Lys318Asn)	M	-	Sleep-related tonic–clonic; focal impaired awareness seizures; tonic seizures	Epileptic activity from left fronto-lateral	Valproate, levetiracetam, brivaracetam/carbamazepine, clobazam	Pharmo resistance, NVS implanted	DD, behavioral issues, ASD, ADHD	NA	Parenti et al. (6)
77	c.614 T > A (p.Leu205Gln)	M	Defecation	-	Focal slow spike-waves (left frontal and temporal lobes)	Lamotrigin	Seizure-free	DD, behavioral issues	NA	Parenti et al. (6)
78	c.774G > T (p.Met258Ile)	M	Hyperpnea	-	Diffuse slow spike-waves	-	-	DD, behavioral issues	NA	Parenti et al. (6)
79	c.745C > T (p.Gln249*)	M	NA	-	Normal	NA	NA	DD, behavioral issues, ASD	NA	Parenti et al. (6)
80	c.340A > G (p.Arg114Gly)	M	Stroboscope	Febrile and non-febrile seizures.	Nap sleep that appears imperfectly organized (hypnic figures rare and sometimes difficult to individualize) but devoid of peak figures.	LVT	NA	DD, ID, behavioral issues	NA	Parenti et al. (6)
81	c.39del (p.Phe13Leufs*10)	M	Toothbrushing, warm bath or shower	Epilepsy	Normal	ESL since summer 2020 to February 2021, switched to CBZ (Tegretol R).	Pharmo resistance	ID	NA	Parenti et al. (6)
						July 2021: switch from CBZ to ZNS.				
						September 2021: ZNS + CBZ (current treatment)				
82	c.1121C > T (p.Ala374Val)	M	NA	-	NA	NA	NA	DD, behavioral issues, ASD, ADHD	NA	Parenti et al. (6)
83	c.1121C > T (p.Ala374Val)	M	NA	-	NA	NA	NA	DD, ID, behavioral issues, ASD	NA	Parenti et al. (6)
84	c.980 + 43_981del (p.Met327Ilefs*81)	F	NA	-	NA	NA	NA	DD, ID	NA	Parenti et al. (6)
85	c.614_616dup (p.Leu205dup)	M	Warm bath	Only generalized tonic–clonic. Once he had an hemiclonic seizure, Febrile seizures	Multifocal epileptiform activity (predominantly frontal), background slowing	Current_CLB, ZNS, LMT, ESL. Previously LEV, VPA, PHT, PER	Pharmo resistance	DD, ID	NA	Parenti et al. (6)
86	c.528-2A > T (p.?)	M	Contact with water	Atonic	NA	Lamotrigine	Seizure-free	ID, behavioral issues, ADHD	NA	Parenti et al. (6)
87	c.32G > C (p.Ser11Thr)	F	NA	Seizures or encephalopathy	NA	NA	NA	NA	NA	van der Ven et al. (25)
88	c.1666C > T (p.Arg556Cys)	F	NA	Epilepsy	NA	NA	NA	DD	NA	Yang et al. (28)
89	c.376 T > A (p.Trp126Arg)	M	NA	Epilepsy	NA	NA	NA	DD	NA	Fernández-Marmiesse et al. (24)
90	c.718G > A (p.Gly240Arg)	F	NA	Epilepsy	NA	NA	NA	NA	NA	Fernández-Marmiesse et al. (24)
91	c.1648G > A (p.Ala550Thr)	F	NA	NA	NA	NA	NA	Mild ID, behavioral issues	NA	Mojarad et al. (29)
92	c.477_479delTGG (p.Gly160del)	M	-	Focal epilepsy with secondary generalization	Generalized slow waves	VPA	Seizure-free	Behavioral abnormalities	NA	Leuschner et al. (30)
93	c.477_479delTGG (p.Gly160del)	M	-	Focal epilepsy with secondary generalization	Bioelectrical status epilepticus with irregular generalized spike–wave discharges	VPA	Seizure-free	ADHD	NA	Leuschner et al. (30)

NA, not assessed; F, female; M, male; ASD, autistic spectrum disorder; ADHD, attention deficit hyperactivity disorder; TCS, Tonic-clonic seizures; GTCS, Generalized tonic-clonic seizure; GDD, global developmental delay; EEG, Electroencephalogram; DD, developmental delay; ID, intellectual disability.

A meticulous re-evaluation of the pathogenicity of 23 reported non-truncating variants in 31 patients unveiled that most reported missense variants could be appropriately categorized as Variants of Uncertain Significance (VUS) by the ACMG guidelines (14, 23, 24) (Tables 1, 2). According to ClinGen recommendations for PP3/BP4 criteria updated in 2022, we relied on the REVEL score to provide supporting, moderate, and strong levels of evidence for pathogenicity or benignity (31). Functional studies indicated that *SYN1* missense variants, such as Ser79Trp, Arg420Gln, Ala550Thr, and Thr567Ala, may exert an impact on neuronal development and nerve terminal targeting. These variants were associated with the disruption of synaptic vesicle pools and an increased frequency of excitatory spontaneous release events (8–10). However, it is noteworthy that the Thr567Ala variant was detected in 755 hemizygous and 48 homozygous healthy carriers in gnomAD v4.0.0. The high allele frequency implies a proclivity toward benign polymorphism rather than a pathogenic variant. Ala51Gly was also noted with high allele frequency in gnomAD v4.0.0 with 670 hemizygous and 42 homozygous healthy carriers, whereas Gly240Arg shows an incongruent pattern of inheritance (32). The three variants (Thr567Ala, Ala51Gly, Gly240Arg) tend to be benign. Uncertainty persists regarding the functional implications of other non-truncating variants, considering the limited scope of *in vitro* experiments conducted thus far. The question of whether *SYN1* missense variants significantly contribute to *SYN1*-related disease remains elusive in light of these experimental constraints.

**Table 2:** The types and classification of reported *SYN1* gene non-truncating variants.

Variant	REVEL_score	Splice AI	gnomAD (v4.0.0) total allele frequency	ACMG criteria	Classification	Phenotype	References
c.32G > C (p.Ser11Thr)	0.240	0, 0, 0, 0	NA	PM2_Supporting+BP4 + PS2_Supporting+PP2	VUS	Seizures or encephalopathy	van der Ven et al. (25)
c.152C > G (p.Ala51Gly)	0.051	0, 0, 0, 0	2,641/1,165,532, 670 hemi and 42 hom	BA1 + BP4_Moderatre+PP2	Benign	ASD, mild ID	Fassio et al. (9)
c.236C > G (p.Ser79Trp)	0.462	0, 0, 0, 0	NA	PM2_Supporting+PP1 + PS3_Supporting+PP2	VUS	DD, ID	Guarnieri et al. (8)
c.340A > G (p.Arg114Gly)	0.167	0, 0, 0, 0.03	1/439,764, 1 het	PM2_Supporting+BP4_Moderatre+PP2	VUS	Reflex seizures (stroboscope), febrile and non-febrile seizures, DD, ID, behavioral issues	Parenti et al. (6)
c.376 T > A (p.Trp126Arg)	0.594	0, 0, 0, 0	NA	PM2_Supporting+PP2	VUS	Epilepsy, neurodevelopmental delay	Fernández-Marmiesse et al. (24)
c.614 T > A (p.Leu205Gln)	0.780	0, 0, 0, 0	NA	PM2_Supporting+PP3_Moderate+PP2	VUS	Reflex seizures (defecation), DD, behavioral issues	Parenti et al. (6)
c.635G > T (p.Ser212Ile)	0.421	0, 0, 0, 0	NA	PM2_Supporting+PP2	VUS	ASD	Rossi et al. (26)
c.718G > A (p.Gly240Arg)	0.605	0, 0, 0, 0	NA	PM2_Supporting+PP2	VUS	Epilepsy	Fernández-Marmiesse et al. (24)
c.774G > T (p.Met258Ile)	0.355	0, 0.01, 0, 0	NA	PM2_Supporting+PP2	VUS	Reflex seizures (hyperpnea), DD, behavioral issues	Parenti et al. (6)
c.796G > A (p.Val266Met)	0.290	0.02, 0, 0, 0	1/563,600	PM2_Supporting+BP4 + PP2	VUS	mild intellectual disability, autistic features, paranoid schizophrenia	Ibarluzeal. (27)
c.929C > A (p.Ala310Asp)	0.290	0, 0, 0, 0	NA	PM2_Supporting+BP4 + PP2	VUS	Reflex seizures (bathing, hair washing), nocturnal autonomic seizures, febrile seizures	Accogli et al. (2)
c.954G > T (p.Lys318Asn)	0.370	0, 0, 0, 0.11	NA	PM2_Supporting+PS2_Supporting+PP2	VUS	Sleep-related tonic–clonic, focal impaired awareness seizures, tonic seizures, DD, behavioral issues, ASD, ADHD	Parenti et al. (6)
c.986C > T (p.Thr329Met)	0.425	0, 0, 0.04, 0	NA	PM2_Supporting+PS2_Supporting+PP2	VUS	DD, behavioral issues	Parenti et al. (6)
c.1072G > A (p.Asp358Asn)	0.474	0, 0, 0, 0	NA	PM2_Supporting+PP2	VUS	Focal seizures with secondary generalization, DD, behavioral issues, ASD	Parenti et al. (6)
c.1076C > A (p.Thr359Lys)	0.293	0, 0, 0, 0	1/1,089,293, 1 hemi	PP2	VUS	TCS triggered by fever/ febrile seizures, Profound GDD, motor delay, language development delay, social interaction problems	Xiong et al. (4)
c.1121C > T (p.Ala374Val)	0.319	0, 0, 0, 0	4/1,090,684, 4 het	PM2_Supporting+PP1 + PP2	VUS	DD, ID, behavioral issues, ASD, ADHD	Parenti et al. (6)
c.1259G > A (p.Arg420Gln)	0.089	0, 0, 0, 0	5/553,110, 3 hemi and 2 het	BP4_Moderate+PP1 + PS3_Supporting+PP2	VUS	ID from early childhood, mental regression, autistic features	Darvish et al. (10)
c.1648G > A (p.Ala550Thr)	0.153	0, 0, 0.01, 0	0/17,090, 3 hemi and 23 het	BP4_Moderate+PS3_Supporting+PP2	VUS	Idiopathic partial epilepsy, ASD, ID, behavioral issues	Fassio et al. (9) and Mojarad et al. (29)
c.1666C > T (p.Arg556Cys)	0.195	0, 0, 0, 0	2/321,193, 2 het	PM2_Supporting+BP4 + PP2	VUS	Epilepsy, DD	Yang et al. (28)
c.1699A > G (p.Thr567Ala)	0.272	0, 0.01, 0.01, 0	2,718/1,101,774, 755 hemi and 48 hom	BA1 + BP4 + PS3_Supporting+PP2	Benign	ASD, ID	Fassio et al. (9)
c.477_479del (p.Gly160del)	0	0	NA	PM2_Supporting	VUS	Focal epilepsy with secondary generalization, behavioral abnormalities, ADHD	Leuschner et al. (30)
c.614_616dup (p.Leu205dup)	0	0	NA	PM2_Supporting	VUS	Reflex seizures (warm bath), generalized tonic–clonic, once he had an hemiclonic seizure, febrile seizures, DD, ID	Parenti et al. (6)
c.1760_1771dup (p.Arg587_Pro590dup)	0	0	NA	PM2_Supporting	VUS	Reflex seizures (during or after bathing/showering), infantile spasms, 8 months; tonic–clonic seizures with automatism, 2 y; atonic atypical absence seizures, GDD, severe ID, ADHD	Accogli et al. (2)

DD, developmental delay; ID, intellectual disability; ASD, autism spectrum disorder; ADHD, attention deficit hyperactivity disorder, GDD, global developmental delay; het, heterozygotes; hom, homozygotes; hemi, hemizygotes.

## Discussion

This report elucidated the identification of a maternally inherited *SYN1* pathogenic variant in two brothers. Notably, the *SYN1* variant exhibited clinical heterogeneity in both brothers and one previously reported case in the literature (2). A comprehensive review of the literature was undertaken to delineate the prevalence of key clinical features associated with *SYN1*-related disorders, resulting in the compilation of data from a total of 93 documented patients with *SYN1*-associated diseases (Table 1). Among those with *SYN1* truncating variants, 56/62(90.3%) patients reported seizures, and a substantial number of experienced diverse seizure types. In contrast, the non-truncating variant cohort reported only 16/31(51.6%) instances of seizures. These data strongly support the notion that patients with *SYN1* truncating variants are more prone to seizures.

Specifically, in epilepsy patients with *SYN1* truncating variants, 35/56(62.5%) patients reported reflex seizures triggered by activities such as bathing, showering, toothbrushing, rubbing with a towel, fever, fingernail clipping, falling asleep, watching others showering or bathing, and gastrointestinal discomfort (Table 1). In contrast, the non-truncating was 6/16(37.5%) reported instances of seizures, triggered by bathing/showering, defecation, hyperpnea, stroboscope, and hair washing (Table 1). It seems that truncating variants of the *SYN1* gene may tend to manifest REs.

We also analyzed the frequency of spontaneous seizures (non-REs) in epilepsy patients across two groups. The occurrence of non-REs was 21/56(37.5%) in the truncating group and 10/16(62.5%) in the non-truncating group (Table 1). This result further corroborates that reflex seizures may be the primary manifestation in patients with truncating variants. This observation underscores the importance of identifying specific *SYN1* variants to understand unique clinical trajectories, emphasizing the heterogeneity of *SYN1*-related neurodevelopmental disorders. It should also be noted that, although there is a difference between the truncating and non-truncating groups, epilepsy in general is common in patients with *SYN1* variants, with a notable tendency to exhibit reflex epilepsy.

Furthermore, the evaluation of the developmental delay/intellectual disability (DD/ID) phenotype revealed intriguing patterns. A larger proportion of individuals with non-truncating variants 23/31(74%) exhibited DD/ID compared to those with truncating variants 31/62(50%; Table 1). The findings align with previous research (6) but extend our understanding by emphasizing the nuanced relationship between genetic variants and clinical outcomes. However, a deeper understanding of the mechanisms contributing to phenotypic diversity in *SYN1*-related disorders necessitates further investigation.

To date, a total of 81 males and 12 females have been reported with putative *SYN1-*related disorders. Females with *SYN1* variants have exhibited a broad clinical heterogeneity and incomplete penetrance. These female patients have displayed a wide range of clinical manifestations, from simple febrile seizures to severe epileptic encephalopathy, with some individuals presenting DD/ID without seizures (Table 1). Interestingly, epilepsy triggered by bathing was observed in two female patients (Table 1). No differences in variant types were found when compared to male patients, with both truncating and non-truncating variants reported (Table 1). Moreover, two female patients had reported *de novo* variants and two others had reported paternal inherited variants, while somatic mosaicism in the blood was identified in the unaffected father of one of the female individuals (6).

## Conclusion

This report delineates a familial presentation of seizures associated with a pathogenic variant in the *SYN1* gene, thereby broadening the phenotype spectrum of *SYN1* gene-related epilepsy disorders. Through a comprehensive review of existing literature, a discernible pattern emerges wherein truncating variants of the *SYN1* gene tend to manifest in seizure-related symptoms, whereas non-truncating variants are more frequently associated with DD/ID. This discovery may aid in anticipating the phenotypes linked with *SYN1*-related conditions.

Furthermore, upon re-evaluating reported *SYN1* non-truncating variants, a substantial portion of these variants are presently classified as VUS. Consequently, exercising caution is recommended when interpreting *SYN1* missense variants in clinical diagnostics. It is imperative to conduct additional functional studies to definitively ascertain the pathogenicity of these variants.

In summary, our findings contribute valuable insights into the role of the *SYN1* gene in epilepsy disorders, underscoring the necessity for additional research on its genetic variations. These results bear implications for enhancing diagnostic accuracy and providing more informed guidance for patients and their families affected by *SYN1*-related conditions.

## Data availability statement

The datasets presented in this article are not readily available because of ethical and privacy restrictions. Requests to access the datasets should be directed to the corresponding authors.

## Ethics statement

The studies involving humans were approved by the Medical Ethics Committee of Affiliated Hospital of Guilin Medical University. The studies were conducted in accordance with the local legislation and institutional requirements. Written informed consent for participation in this study was provided by the participants’ legal guardians/next of kin. Written informed consent was obtained from the individual(s), and minor(s)’ legal guardian/next of kin, for the publication of any potentially identifiable images or data included in this article.

## Author contributions

BR: Conceptualization, Data curation, Formal analysis, Methodology, Project administration, Resources, Supervision, Writing – review & editing. XW: Data curation, Formal analysis, Investigation, Writing – original draft, Writing – review & editing. YZ: Investigation, Writing – original draft, Writing – review & editing, Formal analysis. LC: Formal analysis, Investigation, Methodology, Project administration, Writing – review & editing, Conceptualization. JJ: Investigation, Methodology, Project administration, Resources, Writing – original draft, Conceptualization.

## Footnotes

^1^ gnomad.broadinstitute.org

## References

[1] Okudan ZV Özkara Ç. Reflex epilepsy: triggers and management strategies. Neuropsychiatr Dis Treat. (2018) 14:327–37. doi: 10.2147/Ndt.S107669 29403278 PMC5779309

[2] Accogli A Wiegand G Scala M Cerminara C Iacomino M Riva A. Clinical and genetic features in patients with reflex bathing epilepsy. Neurology. (2021) 97:E577–86. doi: 10.1212/Wnl.0000000000012298, PMID: 34078716 PMC8424500

[3] Italiano D Striano P Russo E Leo A Spina E Zara F. Genetics of reflex seizures and epilepsies in humans and animals. Epilepsy Res. (2016) 121:47–54. doi: 10.1016/J.Eplepsyres.2016.01.010 26875109

[4] Xiong J Duan H Chen S Kessi M He F Deng X. Familial Syn1 variants related neurodevelopmental disorders in Asian pediatric patients. BMC Med Genet. (2021) 14:182. doi: 10.1186/S12920-021-01028-4, PMID: 34243774 PMC8272254

[5] Nguyen DK Rouleau I Sénéchal G Ansaldo AI Gravel M Benfenati F. X-linked focal epilepsy with reflex bathing seizures: characterization of a distinct epileptic syndrome. Epilepsia. (2015) 56:1098–108. doi: 10.1111/Epi.13042 26096837

[6] Parenti I Leitão E Kuechler A Villard L Goizet C Courdier C. The different clinical facets of Syn1-related neurodevelopmental disorders. Front Cell Dev Biol. (2022) 10:1019715. doi: 10.3389/Fcell.2022.1019715, PMID: 36568968 PMC9773998

[7] Sirsi D Armstrong D Munoz-Bibiloni J Redondo B Jy P. Syn1 gene mutation in a child with focal epilepsy and reflex bathing seizures. J Pediatr Epilepsy. (2017) 6:119–24. doi: 10.1055/s-0037-1599193

[8] Guarnieri FC Pozzi D Raimondi A Fesce R Valente MM Delvecchio VS. A novel Syn1 missense mutation in non-syndromic X-linked intellectual disability affects synaptic vesicle life cycle, clustering and mobility. Hum Mol Genet. (2017) 26:4699–714. doi: 10.1093/Hmg/Ddx352 28973667

[9] Fassio A Patry L Congia S Onofri F Piton A Gauthier J. Syn1 loss-of-function mutations in autism and partial epilepsy cause impaired synaptic function. Hum Mol Genet. (2011) 20:2297–307. doi: 10.1093/Hmg/Ddr122, PMID: 21441247

[10] Darvish H Lj A Tafakhori A Mesias R Ahmadifard A Sanchez E. Phenotypic and genotypic characterization of families with complex intellectual disability identified pathogenic genetic variations in known and novel disease genes. Sci Rep. (2020) 10:968. doi: 10.1038/S41598-020-57929-4 31969655 PMC6976666

[11] Garcia CC Blair HJ Seager M Coulthard A Tennant S Buddles M. Identification of a mutation in Synapsin I, a synaptic vesicle protein, in a family with epilepsy. J Med Genet. (2004) 41:183–6. doi: 10.1136/Jmg.2003.013680 14985377 PMC1735688

[12] Zhou Q Wang J Xia L Li R Zhang Q Pan S. Syn1 mutation causes X-linked Toothbrushing epilepsy in a Chinese family. Front Neurol. (2021) 12:736977. doi: 10.3389/Fneur.2021.736977, PMID: 34616357 PMC8488375

[13] Peron A Baratang NV Canevini MP Campeau PM Vignoli A. Hot water epilepsy and Syn1 variants. Epilepsia. (2018) 59:2162–3. doi: 10.1111/Epi.14572 30390306

[14] Richards S Aziz N Bale S Bick D Das S Gastier-Foster J. Standards and guidelines for the interpretation of sequence variants: a joint consensus recommendation of the American college of medical genetics and genomics and the association for molecular pathology. Genet Med. (2015) 17:405–24. doi: 10.1038/Gim.2015.30, PMID: 25741868 PMC4544753

[15] Sharma A Sankhyan N. Hot-water epilepsy in children. Indian J Pediatr. (2021) 88:857–8. doi: 10.1007/S12098-021-03840-3, PMID: 34227048

[16] Satishchandra P. Hot-water epilepsy. Epilepsia. (2003) 44:29–32. doi: 10.1046/J.1528-1157.44.S.1.14.X 12558829

[17] Yalçin Ad, Toydemir He, Forta H. Hot Water Epilepsy. Clinical and electroencephalographic features of 25 cases. Epilepsy Behav. (2006) 9:89–94. doi: 10.1016/J.Yebeh.2006.03.013 16698323

[18] Mosquera-Gorostidi A Azcona-Ganuza G Me Y-P García De Gurtubay I Aguilera-Albesa S. Ictal video-electroencephalography findings in bathing seizures: two new cases and review of the literature. Pediatr Neurol. (2019) 99:76–81. doi: 10.1016/J.Pediatrneurol.2019.04.017, PMID: 31272783

[19] Meghana A Sinha S Tn S Dk S Satishchandra P. Hot water epilepsy clinical profile and treatment--a prospective study. Epilepsy Res. (2012) 102:160–6. doi: 10.1016/J.Eplepsyres.2012.05.011 22727658

[20] Plouin P Vigevano VF. Reflex Seizures In Infancy. In: P Wolf Y Inoue B Zifkin JL Eurotext, editors. Reflex epilepsies: progress in understanding. Montrouge: John Libbey Eurotext (2004) 115–122.

[21] Braun M Et P Simpkins A Kozlik S Curtis C. Novel bathing epilepsy in a patient with 2q22.3q23.2 deletion. Seizure. (2021) 91:1–4. doi: 10.1016/J.Seizure.2021.05.007 34051608

[22] Franzoni E Gentile V Grosso S Brunetto D Dm C Balestri P. Bathing epilepsy: report of two Caucasian cases. Epileptic Disord. (2010) 12:88–90. doi: 10.1684/Epd.2010.0295, PMID: 20185394

[23] Abou Tayoun AN Pesaran T DiStefano MT Oza A Rehm HL Biesecker LG. Recommendations for interpreting the loss of function Pvs1 Acmg/amp variant criterion. Hum Mutat. (2018) 39:1517–24. doi: 10.1002/Humu.23626 30192042 PMC6185798

[24] Nykamp K Anderson M Powers M Garcia J Herrera B Yy H. Sherloc: a comprehensive refinement of the Acmg-amp variant classification criteria. Genet Med. (2017) 19:1105–17. doi: 10.1038/Gim.2017.37 28492532 PMC5632818

[25] Rosahl TW Spillane D Missler M Herz J Selig DK Wolff JR. Essential functions of Synapsins I and ii in synaptic vesicle regulation. Nature. (1995) 375:488–93. doi: 10.1038/375488a0 7777057

[26] Li L Chin LS Shupliakov O Brodin L Sihra TS Hvalby O. Impairment of synaptic vesicle clustering and of synaptic transmission, and increased seizure propensity, in Synapsin I-deficient mice. Proc Natl Acad Sci USA. (1995) 92:9235–9. doi: 10.1073/Pnas.92.20.9235 7568108 PMC40959

[27] Giannandrea M Fc G Gehring N Monzani E Benfenati F Ae K. Nonsense-mediated Mrna decay and loss-of-function of the protein underlie the X-linked epilepsy associated with the W356× mutation in Synapsin I. PLoS One. (2013) 8:e67724. doi: 10.1371/journal.pone.0067724, PMID: 23818987 PMC3688603

[28] Lewis BP Green RE Brenner SE. Evidence for the widespread coupling of alternative splicing and nonsense-mediated Mrna decay in humans. Proc Natl Acad Sci USA. (2003) 100:189–92. doi: 10.1073/Pnas.0136770100, PMID: 12502788 PMC140922

[29] Chang YF Imam JS Wilkinson MF. The nonsense-mediated decay Rna surveillance pathway. Annu Rev Biochem. (2007) 76:51–74. doi: 10.1146/Annurev.Biochem.76.050106.093909 17352659

[30] Lignani G Raimondi A Ferrea E Rocchi A Paonessa F Cesca F. Epileptogenic Q555x Syn1 mutant triggers imbalances in release dynamics and short-term plasticity. Hum Mol Genet. (2013) 22:2186–99. doi: 10.1093/Hmg/Ddt071, PMID: 23406870 PMC3652419

[31] Pejaver V Byrne AB Feng BJ Pagel KA Mooney SD Karchin R. Calibration of computational tools for missense variant pathogenicity classification and Clingen recommendations for Pp3/Bp4 criteria. Am J Hum Genet. (2022) 109:2163–77. doi: 10.1016/J.Ajhg.2022.10.013 36413997 PMC9748256

[32] Fernández-Marmiesse A Roca I Díaz-Flores F Cantarín V. Pérez-Poyato Ms, Fontalba a, Et Al. rare variants in 48 genes account for 42% of cases of epilepsy with or without neurodevelopmental delay in 246 pediatric patients. Front Neurosci. (2019) 13:1135. doi: 10.3389/Fnins.2019.01135, PMID: 31780880 PMC6856296

